# T315I – a gatekeeper point mutation and its impact on the prognosis of chronic myeloid leukemia

**DOI:** 10.1515/almed-2024-0069

**Published:** 2024-09-16

**Authors:** Bushra Kaleem, Sadaf Shahab, Tahir Sultan Shamsi

**Affiliations:** Department of Clinical Research, National Institute of Blood Disease and Bone Marrow Transplantation, Karachi, Pakistan; Department of Clinical Haematology, National Institute of Blood Disease and Bone Marrow Transplantation, Karachi, Pakistan

**Keywords:** *BCR-ABL*, chronic myeloid leukaemia (CML), disease progression, overall survival, resistance, T315I mutation

## Abstract

**Objectives:**

*BCR-ABL* kinase domain mutations are an important cause of resistance to tyrosine kinase inhibitors (TKIs) in chronic myeloid leukaemia (CML) of which T315I is the most treatment-resilient. This study aimed to observe the frequency of T315I and its impact on disease prognosis in terms of progression and survival.

**Methods:**

Patients with a response which categorized them into warning zone/or who failed to respond to their TKI treatment completely as per the European LeukemiaNet (ELN) were labeled as non-responders. They were assessed for T315I mutation using amplification refractory mutation system-polymerase chain reaction (ARMS-PCR) and validated via sequencing. Patients were then longitudinally followed for 96 months for the prognostic impact of the mutation.

**Results:**

Of the 102 non-responders, T315I mutation was detected in 21.6 % of patients with a female preponderance. Almost 59 % of mutation-harbouring patients were labelled as low Sokal risk at baseline. The disease progression into the blastic phase was reported in 58.8 % of mutation-harbouring patients. Overall survival (study period: 96 months) was 81.8 % in patients harbouring T315I mutation. Patients in the blastic phase had significant odds of harbouring T315I mutation.

**Conclusions:**

Sub-optimal response or failure to TKI treatment indicates the development of resistance due to the presence of T315I mutation or other mutation(s). Early identification will help redirect the patient’s treatment.

## Introduction

Chronic myeloid leukemia (CML) is a myeloproliferative neoplasm that results from a reciprocal translocation t(9;22)(q34.1;q11.2), creating an oncogene that encodes a 210 (kinase domain) kD oncoprotein that enhances cellular proliferative potential [1]. The resultant *BCR-ABL1* oncogene is a diagnostic hallmark in approximately 95 % of CML patients.

Tyrosine kinase inhibitor (TKI) development required a thorough understanding of the pathophysiology of the fusion protein. The fusion protein was intended to be hindered by these compounds specifically. The first-generation inhibitor Imatinib, along with second and third-generation TKIs, changed the way CML is treated. In the International Randomized Study of Interferon vs. STI571 (IRIS), Imatinib demonstrated encouraging outcomes, including an overall survival (OS) rate of 83.3 % and a complete cytogenetic response (CCyR) in 82.8 % of patients. However, around 20 % of patients developed resistance to this medication [2]. Over 90 distinct BCR-ABL kinase domain point mutations were found in these patients. These point mutations prevented Imatinib from binding by interfering with the attachment site or by changing the BCR-ABL’s configuration to a conformation that has a lower affinity for Imatinib [3]. Of all the mutations found, T315I was the most prevalent, accounting for 4–19 % of all mutations that cause resistance in CML patients [4].

Since the more competitive ATP inhibitor of BCR-ABL, Nilotinib, a second-generation TKI, was introduced in 2006, almost 50 % of patients who had demonstrated either primary or secondary resistance to Imatinib treatment achieved better outcomes [4, 5]. All phases of CML showed noticeably improved cytogenetic and hematologic responses [5]. Despite the availability of newer TKIs, some patients continue to show resistance to treatment and mutational analysis has demonstrated that T315I was one of the main causes of resistance. This point mutation results due to the replacement of threonine by isoleucine. The T315I mutation has been reported to be one of the most prevalent mutations in a study of 386 CML patients who had advanced into the blastic phase [6]. In another study comprising of 176 CML patients who received either first- or second-line TKI treatment and harboured the T315I mutation, it was found that patients in the blastic phase of the disease had the lowest survival rates, averaging only 4 months, while patients in the chronic phase of the disease had an overall survival rate of 22.4 months [7]. This study was conducted to evaluate the impact of the presence of the T315I mutation in CML patients on the prognosis of the disease in terms of disease progression and survival.

## Materials and methods

This was a single-centre longitudinal study conducted at the National Institute of Blood Diseases and Bone Marrow Transplantation on the treatment of CML patients receiving TKIs. The study was approved from the Institutional Review Board (NIBD/RD/159-41-2015). Informed voluntary verbal consent from the study participants included in our study was obtained. Patients who displayed primary (failed to gain complete haematological response [CHR] and/or major cytogenetic response [MCyR] at 3 and 6 months respectively) or secondary resistance (progression to advanced disease or loss of already gained response, along with a five to 10 folds increase in *BCR-ABL* transcripts assessed via standardized real quantitative polymerase chain reaction) as per the ELN guidelines at the end of 12 months after the treatment initiation were labelled as non-responders [8]. These non-responders were enrolled consecutively into the study, on whose samples mutational analysis for the presence of T315I was performed via amplification refractory mutation system-polymerase chain reaction (ARMS-PCR) using the primers and thermocycling conditions mentioned in a previous study [9]. The presence of the positive mutation was validated via Sanger sequencing. The patients were observed over an extended period of eight years to ascertain the impact of the mutation on the progression and outcome of the disease, particularly on disease acceleration or enter a blast crisis, or result in death, and to assess the overall survival rate. Patients diagnosed with other Myeloproliferative Neoplasms, or other haematological malignancies, and below the age of 18 years were not included in the study.

### Statistical analysis

The collected data were analyzed using SPSS Version 26.0. After the evaluation of the normality of the data via the Shapiro–Wilk test, the median and interquartile range were computed for quantitative variables while frequency and percentages were used for qualitative variables. Assessment of association was done using Chi-Squared/Fisher Exact test (as per need). The odds of the occurrence of the mutation in relation to various factors were also evaluated. A p-value of <0.05 was considered significant. Kaplan–Meier survival analysis was done for the calculation of the overall survival.

## Results

A total of one hundred and two patients were labelled as non-responders at the time of enrolment. Of these 102 patients, 22 (21.6 %) were found to harbour T315I mutation (Table 1). Of the 22 T315I mutation-harbouring patients, female predominance was observed i.e. 54.5 % (12/22) while males were in majority in the non-mutation-harbouring group. The median (IQR) age in the mutation-harbouring patients was reported to be 37.5 years (31.5–46.5 years) which was considerably lower in comparison to the non-mutation-harbouring group 45.2 years (39.5–60.3 years). Retrospective analysis of the T315I mutation-harbouring patients for the Sokal risk score showed that the majority i.e. 59.1 % had been labelled as low risk followed by intermediate (31.8 %; 7/22), and high risk (9.1 %; 2/22). A similar trend was observed in the non-mutation-harbouring group with the majority of the group in the lower risk. Primary resistance observed in the mutation-harbouring group (5/22; 22.7 %) was higher in comparison to the non-mutation-harbouring group (11/80; 13.8 %).

**Table 1:** Association of various factors with the presence of the mutation.

Variables	Mutation		p-Value
	Present (n=22), n (%)	Absent (n=80), n (%)	
**Gender**			
Male (n=61)	10 (45.5)	51 (63.8)	0.145
Female (n=41)	12 (54.5)	29 (36.2)	
**Resistance**			
Primary (n=16)	5 (22.7)	11 (13.8)	0.328
Acquired (n=86)	17 (77.3)	69 (86.2)	
**Relative Sokal risk score**			
Low (n=65)	13 (59.1)	52 (65.0)	0.792
Intermediate (n=30)	7 (31.8)	23 (28.8)	
High (n=7)	2 (9.1)	5 (6.3)	
**Disease progression**			
No progression (n=40)	5 (22.7)	35 (43.8)	0.051
Progression to AP^a^ (n=35)	7 (31.8)	28 (35.0)	
Progression to BC^b^ (n=27)	10 (45.5)	17 (21.2)	
**Survival status**			
Alive (n=89)	18 (81.8)	71 (88.8)	0.470
Expired (n=13)	4 (18.2)	9 (11.2)	

^a^AP, accelerated phase; ^b^BC, blast crisis.

**Figure 1:**
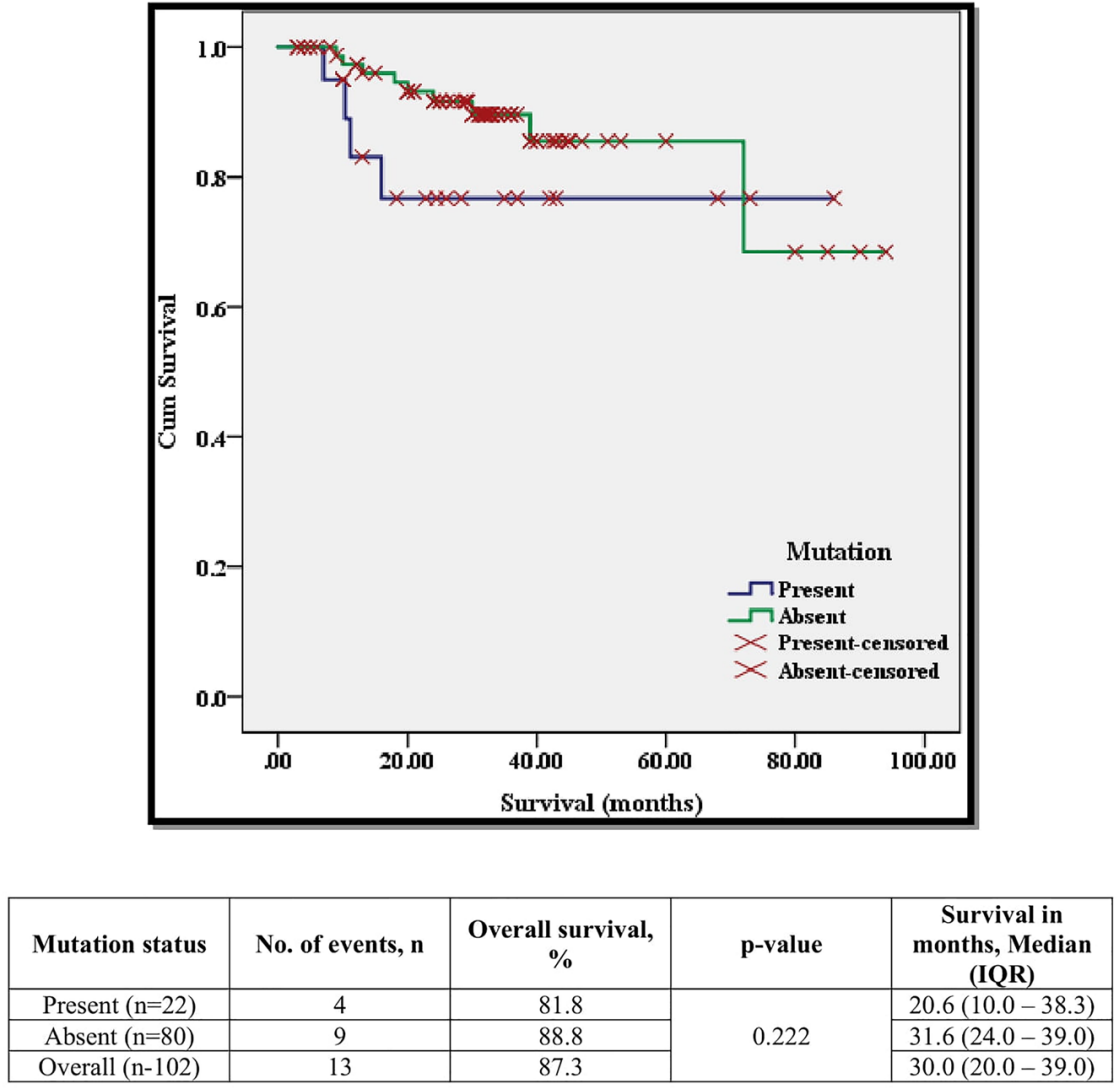
Kaplan–Meier survival analysis of patients based on the presence of T315I mutation.

The progression of the disease i.e. advancement into the accelerated phase or blast crisis, in mutation-harbouring patients, was observed in 17 (77.3 %) patients, out of which 58.8 % (10/17) were found to have progressed to blast crisis. However, in the non-mutation-harbouring group, the disease progression was displayed by 56.3 % (45/80) of which patients with blast crisis were only 37.8 % (17/45). The association of the presence/absence of T315I mutation with various factors is displayed in Table 1. Overall survival observed in an analytical period spanning 96 months, patients harbouring T315I mutation was found to be 81.8 % (time) (Table 1 and Figure 1) in comparison to 88.8 % survival in the non-T315I harbouring group. In terms of survival (months), the median for the mutation-harbouring patients was comparably lower than the non-mutation-harbouring patients (20.6 vs. 31.6 months). The distribution of the patients based on the treatment received, mutation (present vs. absent), and survival status (alive vs. expired) is displayed in Table 2. It was observed that in those patients who harboured the mutation, they either received Imatinib (n=5) or Nilotinib (n=17) with no treatment switch while those without the mutation had the first-line drugs (Imatinib=10 & Nilotinib=51) as well as the treatment switch (Imatinib to Nilotinib=15 & Nilotinib to Imatinib=4). In terms of survival based on the mutation and TKI received, in those who had harboured the mutation, deaths (n=4/17) were only observed in those who received Nilotinib with none in the Imatinib group (n=0/5). Moreover, those who did not harbour the mutation experienced deaths in the Imatinib group (n=1/10), Nilotinib group (n=6/51), and Imatinib to Nilotinib group (n=2/15) with none in the Imatinib to Nilotinib group (n=0/4). Of the 22 patients who harboured the mutation, only four underwent the allogeneic stem cell transplantation of which 3 expired due to transplant-related issues (Graft vs. host disease-3) while one is alive and disease-free.

**Table 2:** Distribution of the patients based on the treatment received, survival, and mutation status.

Mutation	Status	TKI			
		Imatinib (n=5)	Nilotinib (n=17)	Imatinib to Nilotinib (n=0)	Nilotinib to Imatinib (n=0)
Present (n=22)	Alive (n=18)	5 (100.0)	13 (76.5)	–	–
	Expired (n=4)	0	4 (23.5)	–	–

		**Imatinib (n=10)**	**Nilotinib (n=51)**	**Imatinib to Nilotinib (n=15)**	**Nilotinib to Imatinib (n=4)**

Absent (n=80)	Alive (n=71)	9 (90.0)	45 (88.2)	13 (86.7)	4 (100.0)
	Expired (n=9)	1 (10.0)	6 (11.8)	2 (13.3)	0

The odds of the presence of the mutation for various variables are shown in Table 3 which revealed that patients in the blastic phase had significant odds of harbouring T315I mutation (p-value: 0.02).

**Table 3:** Odds ratio of the presence of T315I mutation for various parameters.

Variables	Odds ratio	p-Value	95 % CI
**Gender**			
Female	Ref		
Male	2.110	0.125	0.812–5.484
**Resistance**			
Primary	Ref		
Acquired	1.845	0.310	0.565–6.02
**Relative Sokal risk score**			
Low	Ref		
Intermediate	0.821	0.711	0.289 2.328
High	0.625	0.598	0.109–3.592
**Disease progression**			
No progression	Ref		
Progression to AP^a^	0.571	0.380	0.164–1.996
Progression to BC^b^	0.243	0.023^c^	0.072–0.823
**Survival status**			
Alive	Ref		
Expired	0.570	0.392	0.158–2.065

^a^AP, accelerated phase; ^b^BC, blast crisis; ^c^statistically significant.

## Discussion

Imatinib transformed the treatment of CML, turning a lethal malignancy into a condition that could be managed. Subsequently, it became evident that a favorable treatment outcome required constant long-term drug exposure in order to be achieved and maintained. Second-line therapy options include higher dosages of Imatinib, a second-generation TKI, or an allogeneic stem cell transplant (allo-SCT) [10]. Second-generation TKIs, such as Dasatinib and Nilotinib, have also demonstrated superior performance to Imatinib in Phase III trials involving newly diagnosed CML, eliciting faster and greater rates of CCyRs and molecular responses in addition to improved overall survival [11, 12].

TKI resistance has emerged as a problem in the treatment of CML, despite significant clinical advancements in the field. Differential drug metabolism and/or drug transport are potential causes of primary resistance, whereas *BCR-ABL* KD mutations, amplification of the *BCR-ABL1* fusion gene, overexpression of drug transporter genes, and overexpression of tyrosine kinases like SRC are potential causes of acquired resistance [13, 14]. As reported in 35–45 % of patients, KD mutations are the most recurrent source of acquired resistance. According to Indian studies, 45–50 % of patients who exhibit Imatinib resistance have mutations [15, 16]. 41 percent of the samples in a comprehensive research with data from over a thousand patients from different parts of India had mutations [17]. According to a study, the reported proportion of the mutation is also dependent on the stage of the disease at which the analysis was conducted. Patients with more advanced diseases may have a higher frequency of these mutations [15].

The “gatekeeper mutation,” or T315I, is one of the most frequent mutations. Its treatment is considered a challenger because Imatinib as well as two second-generation TKIs, Dasatinib and Nilotinib, are ineffective against it [18]. Similar to an Indian study, ours revealed that the mutation T315I was present in 21.6 % of all non-responders [17]. This percentage was higher compared to a study from the West which reported the mutation in 11.8 % of patients [19]. On the other hand, the frequency reported in the present study was lower in comparison to a study by Khair et al. who observed the presence of T315I mutation in 43.4 % of their study cohort [20]. The prediction of the prognosis of patients was done utilizing the Sokal score at the time of diagnosis. The Sokal score was calculated in our study cohort and reported 59.1 % of patients to have a low-risk score in T315I mutation harbouring patients followed by intermediate risk (31.8 %), and high-risk score (9.1 %) (Table 1). Our finding followed the descending pattern of the frequency of patients as the risk increased as reported by Hasford et al. [21].

Clones that possess mutations in their kinase domain might exhibit distinct biological characteristics that could increase their susceptibility to leukemia compared to clones without such mutations [22]. Additionally, various studies have shown that individuals with the T315I mutation typically experience more severe clinical outcomes than those with other mutations or those who acquire Imatinib resistance through other means [23]. This fact was shown by a study from the China which reported that 68.4 % (13/19) of their CML patients harbouring a T315I mutation did not respond to Imatinib dose escalation and progressed to an advanced phase [24]. A similar finding was also observed in our study as the mutation-positive group showed advancement of the disease in 77.3 % (17/22) of our study participants (Table 1), of which 58 % progressed to the blastic phase. It was further observed that there were 0.24 times more chances of developing the mutation if the disease was in the blastic phase (p-value: 0.023) in comparison to the disease being in the chronic or accelerated phase (Table 2). Another study from Egypt showed that the frequency of mutations was higher in advanced phases i.e. accelerated and blast phases (10/11, 91 %) compared to chronic phase (CP) (6/17, 35 %) indicating a causal relationship between disease progression and development of mutation [25].

The overall survival (in a study period of 96 months) in the present study of mutation-harbouring patients was found to be 81.8 % in comparison to 88.8 % survival in non-T315I mutation-harbouring patients (Table 1). However, no statistical difference was observed in terms of survival and the presence of the mutation. Jabbour et al. reported overall survival of 59.3 % in T315I mutation-harbouring patients in comparison to 61.6 % in non-T315I mutation-harbouring patients [23].

The percentage of the mutation carriers seems to vary depending on the methodology used, such as the allele-specific PCR (sensitivity: 0.01 %), denaturing high-performance liquid chromatography (sensitivity: 0.1–10 %) or sequencing (sensitivity: 10–20 %) [9]. So in comparison to various other techniques, ASO-PCR could be considered a rapid and inexpensive technique for the detection of the mutation for screening purposes. However, since sequencing is considered a confirmatory test for any screening method, it should be recommended to perform sequencing if the mutation is initially detected via ASO-PCR. This line of action was used in the current study and should especially be recommended in the presence of T315I mutation which can increase the leukemogenic potential of the *BCR-ABL* and is also resistant to all available first, second, and third-generation TKIs except Ponatinib which is unavailable in many countries.

Dasatinib and Nilotinib, two second-line TKIs that are effective against many clinically important mutations for which Imatinib is ineffective, did not affect the BCR-ABL T315I mutation. The Phase II Ponatinib Ph+ALL and CML Evaluation (PACE) trial showed that Ponatinib was successful in achieving major cytogenetic response (MCyR) in 56 % of patients with chronic phase CML and complete cytogenetic response (CCyR) in 46 % of patients with Dasatinib or Nilotinib resistance or patients with the T315I mutation [26]. However, Pakistan does not have access to the medication.

One of the limitations of the present study was our inability to monitor the cytogenetic and molecular response in our patients at pre-determined landmarks. As Ponatinib is yet not available in Pakistan, the patients who were found to harbour T315I mutation, have either been offered cytoreductive therapy or the option of bone marrow transplantation which is an expensive procedure.

## Conclusions

Under the ELN recommendations, mutational analysis is a critical step that needs to be undertaken in case of the presence of primary or secondary resistance. Before shifting to a second-line TKI treatment, the mutational status of the patient should be assessed. The presence of a mutation helps in tailoring the subsequent treatment of the patient. Regular monitoring is key to overcoming resistance and also achieving optimal responses. As the third line of treatment (i.e. Ponatinib) is yet not available in Pakistan, the patients have either been offered cytoreductive therapy or the option of bone marrow transplantation. There is also a need for proper implementation of the performance of mutational analysis before shifting to another TKI to prevent exposure to the drugs resulting in patients either being categorized into the warning zone or who showed no response at all.
